# HTLV-1 Infection and Cervicovaginal Susceptibility to High-Risk HPV: Findings from Women Living with HTLV-1 in Salvador, Brazil

**DOI:** 10.3390/v17020140

**Published:** 2025-01-22

**Authors:** Alisson de Aquino Firmino, Paulo Roberto Tavares Gomes Filho, Juliana Domett Siqueira, Luana Leandro Gois, Giselle Calasans de Souza Costa, Adenilda Lima Lopes Martins, Mariana Lima Drumond, Marcelo Alves Soares, Bernardo Galvão-Castro, Carlos Gustavo Régis da Silva, Maria Fernanda Rios Grassi

**Affiliations:** 1 Centro de Atendimento ao Portador de HTLV (CHTLV), Escola Bahiana de Medicina e Saúde Pública (EBMSP), Salvador 40290-000, BA, Brazil; 2 Centro de Pesquisa (CPQ), Instituto Nacional de Câncer (INCA), Rio de Janeiro 20231-050, RJ, Brazil; 3 Laboratório Avançado de Saúde Pública (LASP), Instituto Gonçalo Moniz, Fundação Oswaldo Cruz (FIOCRUZ), Salvador 40296-710, BA, Brazil; 4 Instituto de Ciências da Saúde (ICS), Universidade Federal da Bahia (UFBA), Salvador 40110-902, BA, Brazil; 5 Departamento de Saúde (DSAU), Universidade Estadual de Feira de Santana (UEFS), Feira de Santana 44036-900, BA, Brazil

**Keywords:** HTLV-1, HPV, cervical cancer

## Abstract

Persistent oncogenic HPV infection is strongly associated with cervical cancer. Studies have suggested a higher prevalence of HPV in women living with HTLV-1. This study aimed to determine whether HTLV-1 infection is associated with cervicovaginal HPV infection and to characterize HPV types according to oncogenic risk. Vaginal fluid samples were subjected to HPV diagnosis via PCR, and positive samples were subjected to Sanger sequencing and massive sequencing. Papanicolaou smears were examined using light microscopy to identify cell abnormalities. Among the 155 women screened, 79 were HTLV-1-infected and 76 were uninfected. HPV PCR identified 23 positive samples (15/79 vs. 8/76; *p* = 0.13). Twenty-three HPV types were identified, of which only types 31, 54, and 58 were present in both groups. When the number of HPV58 infections in each group was compared, women with HTLV-1 had a higher prevalence (8/79 versus 1/76; *p* = 0.03). In total, 61.9% of HTLV-1-infected women had at least one high-risk or probable high-risk HPV type (*p* = 0.12). Cytopathological findings were not significantly different between the groups. Further research is needed to determine whether HTLV-1 infection affects HPV progression and cervical cancer development and to assess the potential benefits of vaccination for women living with HTLV-1.

## 1. Introduction

Human T-cell leukemia virus type 1 (HTLV-1) is a retrovirus that is transmitted through contact with contaminated blood, from mother to child during pregnancy, or primarily through breastfeeding and sexual contact [1,2]. It is estimated that 5–10 million individuals are infected with this virus globally, with a significant prevalence in Brazil, where approximately 800,000 individuals are infected [3]. Salvador exhibits a general population prevalence of 1.8%, with a higher prevalence in females (2.0%) than in males (1.2%). The prevalence of HTLV-1 increases with age, particularly in females, reaching 10% in those aged > 50 years [4]. A recent study demonstrated that sexual transmission between adults is the primary route of HTLV-1 infection in the general population of Salvador [5].

Another virus of significant concern is human papillomavirus (HPV), which is the most prevalent sexually transmitted infection (STI) globally [6,7,8,9,10]. To date, more than 200 distinct HPV types have been identified, which are classified as low- or high-risk based on their association with cervical cancer [11,12]. High-risk HPV types, including HPV16 and -18, are responsible for approximately 90% of invasive cervical cancers, whereas low-risk HPV types, such as HPV6 and -11, are primarily associated with benign lesions, including genital warts and laryngeal papilloma [13,14,15,16]. It is estimated that a minimum of 50% of sexually active individuals will be exposed to HPV at some point in their lives, and by the age of 50, approximately 80% of women will be exposed to the virus [17,18].

Despite the recognized global burden of HPV and its role in cervical cancer, the epidemiology of HTLV-1 and HPV coinfection remains poorly characterized. A limited number of studies have investigated the interaction between these two viruses in the cervicovaginal environment [19,20,21]. A pilot study of 90 women demonstrated a higher prevalence of HPV among patients infected with HTLV-1 [19], while another study reported that HTLV-1 infection is associated with high-risk HPV infection, with HPV16 being the most prevalent type [20]. Conversely, a study that examined the association between 24 specific types of HPV and HTLV-1 infection identified an association solely with HPV53, a probable high-risk type [21].

Our previous study demonstrated that HTLV-1 proviral load is detectable in the vaginal fluid and that viral infection induces local immunological activation, as evidenced by elevated levels of Th1, Th2, and IL-17 cytokines. Furthermore, we observed that women with HTLV-1-associated myelopathy/tropical spastic paraparesis (HAM/TSP) exhibited decreased vaginal lubrication compared with asymptomatic HTLV-1 carriers and uninfected women. This finding suggests that the combination of a more inflamed vaginal environment and reduced natural lubrication may increase the susceptibility of HTLV-1-infected women to other STIs, including HPV [22,23,24]. Given the oncogenic potential of HPV and the dysregulation of the immune system caused by HTLV-1 infection, elucidating the interactions between these viruses is of paramount importance.

Investigating whether HTLV-1-infected women exhibit increased HPV infection rates and identifying the types of HPV present, particularly those with high oncogenic risk, could provide valuable insights into the copathogenesis of these viruses and their impact on the cervicovaginal microenvironment. Furthermore, this knowledge could contribute to the development of improved prevention and treatment strategies, particularly for populations at risk of coinfection. This study aimed to determine whether HTLV-1 infection is associated with cervicovaginal HPV infection in women and to characterize HPV types according to oncogenic risk.

## 2. Materials and Methods

### 2.1. Patients and Study Design

This cross-sectional study was conducted at the Integrative Multidisciplinary HTLV Center (CHTLV) of the Bahiana School of Medicine and Public Health (EBMSP) in Salvador, Bahia, Brazil, from October 2014 to November 2015 [25]. Patients were included sequentially at the time of consultation based on the following inclusion criteria: diagnosis of HTLV-1 infection (enzyme-linked immunosorbent assay and Western blot-positive), age > 18 years, and active sexual life. The uninfected group was selected from companions or relatives of patients who had attended consultations using the same criteria, such as age and sexual activity. Women who met one of the following criteria were excluded from the study: positive HIV serology; vaccinated against HPV; pregnant; postpartum up to 42 days; breastfeeding; transplant recipient; undergoing chemotherapy, radiotherapy, or corticosteroid therapy; suffering from a disease that affects the immune system; and undergoing total hysterectomy or cervical amputation.

The sample size was calculated based on a 30% estimated prevalence of HPV infection in HTLV-1-uninfected women and 60% in HTLV-1-infected women, with an estimated prevalence ratio (PR) of 2.0 [17,19]. Adopting an alpha error of 5% and a power of 80%, the necessary sample size was determined to be at least 49 women in each group.

### 2.2. Ethical Approval

The study protocol was approved by the EBMSP Institutional Research Board on 24 September 2014 (CAAE 33098414.4.0000.5544). All the procedures were designed and implemented in accordance with the ethical principles outlined in the Declaration of Helsinki [26]. Written informed consent was obtained from all participants prior to their participation in the study.

### 2.3. Sample Collection

Clinical and demographic data were obtained using a standardized form. Whole blood samples were procured in EDTA tubes, and peripheral blood mononuclear cells (PBMCs) were isolated by density gradient centrifugation and cryopreserved until use. A single trained gynecologist (PRTGF) conducted a comprehensive gynecologic examination and collected cervicovaginal samples. Cotton swabs were used to obtain fluids from the ectocervix, endocervix, and vaginal walls for molecular HPV diagnosis. The samples acquired for analysis were subsequently placed in tubes containing 400 µL of hydroxymethyl ethylenediamine tetra-acetic acid (Tris-EDTA) solution and stored at −20 °C. For cytopathological and vaginal microbiota analyses, Papanicolaou smears were obtained from the ectocervix and endocervix using an Ayres spatula and a cytobrush, respectively. The collected samples were fixed in absolute alcohol before further processing.

### 2.4. Cytopathological Analysis and HPV Genotyping

Cell abnormalities detected by Papanicolaou smears were classified according to the Bethesda system using light microscopy [27]. Total DNA was extracted with the QIAamp DNA mini kit (Qiagen, Valencia, CA, USA) using the Spin Column DNA Extraction System and stored at −20 °C until use. The presence of HPV DNA was determined by PCR using the degenerate primers MY09 (5′-CGTCCMARRGGAWACTGATC-3′) and MY11 (5′-GCMCAGGGWCATAAYAATGG-3′), which were annealed in a conserved region of the L1 gene and amplified a product of approximately 450 bp [28]. In another assay, PC04/GH20 primers were incorporated, which amplified a 268 bp cellular β-globin DNA fragment that served as an internal control [29]. The standard PCR reaction mix was adjusted to a final volume of 25 µL using the following reagent concentrations: NZYTaq II 2× Green Master Mix (NZYTech, Lisbon, Portugal), MY09/MY11 (6.5 pmol of each primer) or PC04/GH20 (6 pmol of each primer), and 100 ng of sample DNA. A previously HPV-diagnosed sample provided by a partner laboratory served as the positive control. Ultrapure water without DNA was used as the negative control. Fragments were amplified in a thermocycler under the following conditions: initial denaturation at 94 °C for 4 min; 40 cycles of denaturation at 94 °C for 30 s, annealing at 55 °C for 30 s, and extension at 72 °C for 30 s; and final extension at 72 °C for 8 min. The PCR products were subjected to electrophoresis on 1% agarose gel under a constant voltage. The bands were visualized by staining with SYBR Safe DNA Gel Stain (Invitrogen, Waltham, MA, USA) and photographed. Positive PCR results were obtained by Sanger sequencing using a 3500XL Genetic Analyzer (Applied Biosystems, Waltham, MA, USA).

### 2.5. Circular DNA Enrichment, Sequencing, and HPV Complete Genome Analysis

Circular DNA was enriched by rolling-circle amplification (RCA) using the Illustra TempliPhi Amplification Kit (GE Healthcare Life Sciences, Piscataway, NJ, USA) in accordance with the manufacturer’s protocol. The RCA products were visualized by 0.8% agarose gel electrophoresis as a band approximately 12 kb in size. DNA products were purified, and 2 ng was used to prepare each sample library using the Nextera XT DNA Sample Preparation Kit (Illumina Inc., San Diego, CA, USA). All samples were individually indexed through the addition of pairs of indexing primers, and library concentrations were determined using a Qubit 4 fluorometer (Thermo Fisher Scientific, Wilmington, DE, USA). Libraries were sequenced on an Illumina MiSeq platform (2 × 300 nt reads). Reads with a Phred quality score below 28 were trimmed, and the remaining reads were assembled with HPV reference genomes from the Papillomavirus Episteme (PaVE) database using Geneious R11 (Biomatters, Auckland, New Zealand). Assemblies were verified manually, and samples were considered positive for a given HPV type based on previously defined criteria [30]: presence of one read properly mapped to L1 ORF or presence of two or more reads properly mapped to different regions of the reference genome. Consensus sequences covering more than 95% of the genomes were extracted and classified into HPV-type lineages or sublineages according to the identity between the assembled genomes and reference sequences calculated using Geneious R11 [31].

### 2.6. Data Availability

Sequencing data files generated in this study were submitted to the Sequence Read Archive (SRA) database and are available under project number PRJNA1194018. HPV complete genomes obtained were deposited in the GenBank database and assigned the accession numbers PQ720434 to PQ720440.

### 2.7. Statistical Analysis

Quantitative sociodemographic and clinical variables without a normal distribution, such as age, educational level, weekly sexual frequency, number of partners, pregnancies, parity, and abortions, were analyzed using the nonparametric Mann–Whitney U test and presented as median values and 25th and 75th percentiles. Qualitative variables of skin color, marital status, smoking, social alcohol use, illicit drug use, sexually transmitted infections, treatment with trichloroacetic acid, dyspareunia, bleeding after sexual intercourse, and condom use were expressed as simple frequencies/proportions and analyzed using the chi-squared test or Fisher’s exact test. HPV PCR was performed using the chi-square test. HPV oncogenic risk and differences in cervicovaginal cytopathological profiles were assessed using Fisher’s exact test. Statistical significance was set at *p* ≤ 0.05. All analyses were performed using the GraphPad software (version 9.5) and SPSS software (version 17.0) for Windows.

## 3. Results

A total of 155 women were examined, of whom 79 were infected with HTLV-1 and 76 were uninfected. No significant differences were observed between the groups in sociodemographic profiles or substance use (Table 1). HTLV-1-infected women demonstrated distinct sexual behaviors, reporting lower weekly sexual frequency (*p* < 0.0001), a greater number of lifetime sexual partners (*p* = 0.0029), and fewer sexual partners in the past six months (*p* = 0.0007). HTLV-1-infected women had a higher rate of pregnancies and births (*p* < 0.0001); however, there was no significant difference in the number of abortions between the groups (*p* = 0.86).

Regarding the clinical characteristics, no significant disparities were found in sexually transmitted infection history, trichloroacetic acid use for HPV treatment, dyspareunia, or post-intercourse bleeding. However, HTLV-1-infected women reported lower condom use than uninfected women did (*p* = 0.05) (Table 1).

Cytopathological analysis revealed no significant differences in atypical or lesion frequencies between HTLV-1-infected and -uninfected women (Table 2). Six cases exhibited atypia or lesions, with three cases (1 ASC-US, 1 LSIL, and 1 HSIL) in the HTLV-1-infected group and three cases (1 ASC-US and 2 LSIL) in the HTLV-1-uninfected group. HPV PCR testing of vaginal samples identified 23 positive cases, comprising 15 (19%) HTLV-1-infected women and 8 (10.5%) uninfected women (15/79 vs. 8/76; *p* = 0.13) (Table 2).

Among the 23 HPV PCR-positive samples, only 3 demonstrated atypical findings in cervicovaginal cytopathology: one ASC-US in an HTLV-1-infected individual and two LSILs in uninfected individuals. All PCR-positive samples underwent Sanger sequencing and next-generation sequencing (NGS), resulting in the identification of HPV types in 13 HTLV-1-infected individuals and 7 uninfected individuals. Circular DNA NGS detected HPV in 12 samples and identified four individuals with multiple HPV types, two in the HTLV-1-infected and two in the HTLV-1-uninfected group (Table 3).

**Figure 1:**
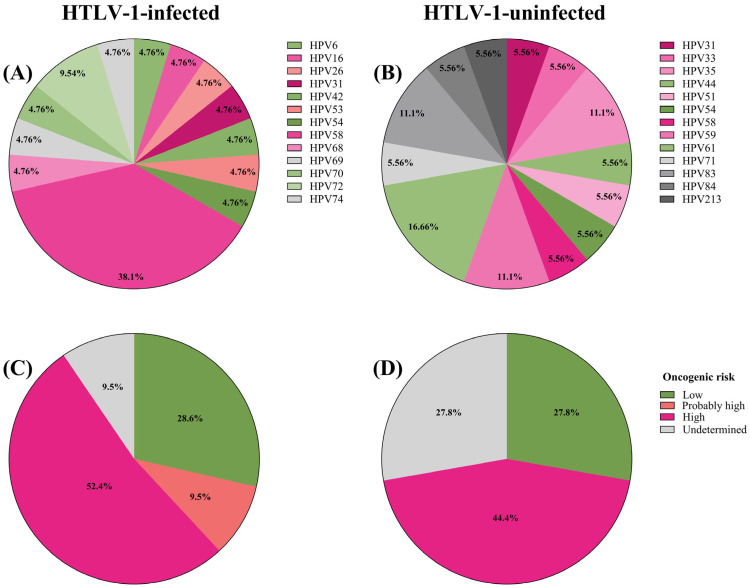
Percentage distribution of HPV types found by Sanger sequencing and NGS in HTLV-1-infected (*n* = 13) and -uninfected (*n* = 7) women (**A**,**B**), along with the percentages of oncogenic risk: 61.9% of HTLV-1-infected women harbored at least one high-risk or probable high-risk HPV type (HPV16 (*n* = 1), HPV26 (*n* = 1), HPV31 (*n* = 1), HPV53 (*n* = 1), HPV58 (*n* = 8), or HPV68 (*n* = 1)) compared to 44.4% of uninfected women (HPV31 (*n* = 1), HPV33 (*n* = 1), HPV35 (*n* = 2), HPV51 (*n* = 1), HPV58 (*n* = 1), or HPV59 (*n* = 2)) (**C**,**D**). Data were analyzed using frequencies/proportions and Fisher’s exact test (*p* = 0.12). Analysis was performed using GraphPad Prism software (version 9.5; San Diego, CA, USA).

A total of 23 HPV types were identified by sequencing. Thirteen HPV types were detected in HTLV-1-infected women, and thirteen types were found in uninfected women, with only three types (HPV31, HPV54, and HPV58) present in both groups. Regarding the percentage distribution of HPV types among HTLV-1-infected women, HPV58 was the most frequent (38.1%), followed by HPV72 (9.54%), and all other identified types accounted for 4.76%. Among HTLV-1-uninfected women, HPV61 was the most frequent type (16.66%), followed by HPV35, HPV59, and HPV83 (11.1%), whereas other types accounted for 5.56% (Figure 1A,B). When the overall prevalence of HPV58 infections was compared between groups, HTLV-1-infected women exhibited a prevalence of 10.1%, which was significantly higher than the 1.3% observed in uninfected women (8/79 vs. 1/76; *p* = 0.03). Among HTLV-1-infected women, 61.9% harbored at least one high-risk or probable high-risk HPV type: HPV16 (n = 1), HPV26 (n = 1), HPV31 (n = 1), HPV53 (n = 1), HPV58 (n = 8), or HPV68 (n = 1), compared to 44.4% of uninfected women: HPV31 (n = 1), HPV33 (n = 1), HPV35 (n = 2), HPV51 (n = 1), HPV58 (n = 1), or HPV59 (n = 2) (Figure 1C,D) [13]. However, this difference was not statistically significant (*p* = 0.12).

Seven complete HPV genomes comprising six different HPV types were assembled from six samples: HPV58 (*n* = 2), HPV16 (*n* = 1), HPV26 (*n* = 1), HPV54 (*n* = 1), HPV69 (*n* = 1), and HPV72 (*n* = 1). Among these, HPV72 has not been established, and the complete assembled genome showed high identity (99.26%) to the reference genome. The remaining six genomes were classified as one of the previously described sublineages. Both genomes from HPV58 were from lineage A2, the HPV16 genome belonged to lineage A3, HPV26 belonged to lineage A, HPV54 belonged to lineage A2, and HPV69 belonged to lineage A4.

## 4. Discussion

The results of the present study demonstrated that the frequency of HPV in the cervicovaginal fluid of HTLV-1-infected women was approximately twice that of HTLV-1-negative women, although the difference was not statistically significant owing to the small number of total and HPV-positive samples. Furthermore, this study revealed that more than half of the HTLV-1-infected women harbored one or more HPV types with a high or probable high oncogenic risk. Remarkably, HPV58, a high-risk virus, was detected in 10.1% of HTLV-1-infected women, whereas its prevalence in the HTLV-1-uninfected group was only 1.3%. HPV58 is associated with persistent high-grade lesions and a high prevalence of cervical cancer. A study conducted in China reported that one-third of patients with cervical cancer were positive for HPV58 [32,33]. A meta-analysis evaluating HPV types globally by region showed that HPV58 is one of the most common types in Latin America, along with HPV16, HPV18, HPV31, and HPV52 [34]. In Salvador, Brazil, studies conducted among women living with HIV and in the general population also demonstrated a notable prevalence of HPV58, which may account for its high frequency in the HTLV-1-infected women found in the present study [35,36]. A noteworthy observation in both HTLV-1-infected and -uninfected women was the presence of coinfection with multiple HPV types, which was observed in four patients and has been primarily reported in individuals living with HIV [37,38,39]. However, HIV seropositivity was an exclusion criterion in this study.

To the best of our knowledge, only a limited number of studies have conducted a more comprehensive analysis of coinfection, and there is no consensus regarding the epidemiological aspects of the HTLV-1/HPV relationship [19,20,21]. A study conducted in the Peruvian Amazon region reported that HTLV-1 infection is associated with high-risk HPV infections. The most prevalent high-risk HPV type was HPV16 (10.8%), followed by HPV31 (5.9%) and HPV18 (4.9%) [20]. An additional study conducted in Kenya examined the association between 24 specific types of HPV and HTLV-1 infection and identified an association in only 1 type, HPV53, which is classified as a probable high-risk type [21]. Although not statistically significant, a higher proportion of HPV infections was observed in HTLV-1-infected women (19% vs. 10.5%). These findings corroborate the results of our previous study conducted by our team in a different sample of patients, which also reported a twofold-higher percentage of HTLV-1/HPV-coinfected women (44% vs. 22.5%) compared to women without HTLV-1 exposure who tested positive for HPV [19]. Comparable results were obtained in another study, which indicated that women with HTLV-1 were twice as likely to have HPV infection of any type than HTLV-1-negative women [20]. Notably, in the present study, women infected with HTLV-1 had a higher number of lifetime sexual partners and lower rates of condom use. Only 2.5% of women reported consistent condom use. Although vaccines remain the most effective means of protection against HPV, consistent condom use has been shown to significantly reduce the risk of HPV infection [40,41].

Consistent with previous studies, the present investigation did not observe a significant difference in cervicovaginal cytopathological analysis between HTLV-1-infected and -uninfected individuals [19,20,21,22]. Over 90% of the women in both groups were diagnosed as negative for intraepithelial lesion or malignancy (NILM). The cytological findings of only three patients corresponded to positive PCR and HPV genotyping results obtained using Sanger sequencing or NGS. Even in a patient with HSIL, a precursor lesion of squamous cell carcinoma [42], the results were not corroborated by PCR. Furthermore, 17 cases with NILM cytopathology were positive by PCR and confirmed by NGS or Sanger sequencing. Multiple studies have demonstrated that cytological findings have a low sensitivity for the diagnosis of HPV infection [43,44,45]. As reported in the literature, HPV infection of the cervix is strongly correlated with cervical cancer. Approximately 99.7% of squamous cell carcinomas are caused by persistent high-risk genital HPV infection, and nearly 90% of cervical adenocarcinomas are associated with the presence of the virus [8,46,47].

Regarding HTLV-1 infection, there is no consensus among studies on its association with cervical cancer. A study conducted in Japan suggested that HTLV-1 infection may influence the oncogenic prognosis of certain patients with cervical or vaginal cancer [48]. Consistent with this finding, a study in Jamaica demonstrated increased rates of HTLV-1 infection in individuals with high-grade cervical intraepithelial neoplasia or cancer compared to those with low-grade cervical intraepithelial neoplasia or more benign pathological conditions [49]. However, several years later, the same group of researchers obtained data that contradicted their pilot projects. They found that HTLV-1 infection did not significantly contribute to the risk of cervical neoplasia [50]. Two additional studies conducted in Mexico and Kenya concluded that there is no association between HTLV-1 infection and cervical cancer [21,51].

A limitation of this study is the small number of participants, which may have restricted the scope of the investigation regarding both HPV infection and cytopathological alterations. Moreover, longitudinal clinical monitoring of these subjects would be beneficial for observing the persistence of the identified HPV types and their associated risk of carcinogenesis. The pathogenesis of this viral association could be more comprehensively elucidated by quantifying the HTLV-1 proviral load in the vaginal fluid of women coinfected with HTLV-1 and HPV as compared to women infected solely with HTLV-1; however, such quantification was not feasible within the constraints of this study.

## 5. Conclusions

This investigation demonstrated a trend of elevated HPV prevalence among HTLV-1-infected women compared to uninfected controls, although this difference was not statistically significant. While the overall distribution of HPV types was comparable between groups, high-risk HPV type 58 was more prevalent in HTLV-1-infected women. These results suggest a potential association between HTLV-1 and high-risk HPV types, providing a foundation for further investigation into their interaction and the subsequent risk of cervical cancer. Our findings emphasize the need for additional research to evaluate the efficacy of HPV vaccination in women living with HTLV-1 and to inform public health policies regarding immunization strategies for this population.

## Figures and Tables

**Table 1:** Sociodemographic profiles, clinical characteristics, and sexual behavior of HTLV-1-infected and -uninfected women evaluated in Salvador, Brazil.

Variable	HTLV-1-Infected (*n* = 79)	HTLV-1-Uninfected (*n* = 76)	*p*-Value
Age (years) ^1^	39 (26–53)	40 (33–51)	0.78
Educational level (years) ^1^	9 (5–11)	7 (5–10.5)	0.79
Skin color n (%) ^2^			0.74
Black	28 (35.4)	27 (35.5)	
Brown	44 (55.7)	40 (52.7)	
White	7 (8.9)	8 (10.5)	
Indigenous	0 (0)	1 (1.3)	
Marital status n (%) ^2^			0.98
Married/stable union	29 (36.7)	29 (38.2)	
Single	37 (46.8)	35 (46.1)	
Widowed	4 (5.1)	3 (3.9)	
Divorced/separated	9 (11.4)	9 (11.8)	
Smoker n (%) ^3^	5 (6.3)	1 (1.3)	0.21
Social drinker n (%) ^2^	20 (25.3)	11 (14.5)	0.09
Illicit drug user n (%) ^3^	1 (1.3)	1 (1.3)	1.00
Weekly sexual frequency ^1^	0 (0–1)	1 (1–3)	<0.0001
Number of partners ^1^			
Lifetime	3 (1–5)	2 (1–3)	0.0029
Last 6 months	0 (0–1)	1 (1–1)	0.0007
Pregnancies ^1^	3 (1–5)	1 (1–2)	<0.0001
Parity ^1^	2 (1–4)	1 (1–2)	<0.0001
Abortions ^1^	0 (0–1)	0 (0–1)	0.86
STI history n (%) ^3,^*	5 (6.3)	6 (7.9)	0.76
Treatment with TCA n (%) ^3,#^	2 (2.5)	7 (9.2)	0.09
Dyspareunia n (%) ^2^	15 (19)	16 (21.1)	0.90
Bleeding after intercourse n (%) ^3^	4 (5.1)	4 (5.3)	1.00
Condom use n (%) ^3^	2 (2.5)	8 (10.5)	0.05

^1^ Data presented as medians and interquartile ranges (p25–p75); Mann–Whitney U test. ^2^ Data presented as frequencies/proportions; chi-square test. ^3^ Data presented as frequencies/proportions; Fisher’s exact test. * STI: sexually transmitted infections. ^#^ TCA: trichloroacetic acid.

**Table 2:** Frequencies of cervicovaginal cytopathological findings and HPV infection in HTLV-1-infected and -uninfected women.

Variable	HTLV-1-Infected (*n* = 79)	HTLV-1-Uninfected (*n* = 76)	*p*-Value
Cervicovaginal cytopathology n (%) ^1^			
NILM ^a^	74 (93.5)	73 (96.1)	0.72
ASC-US ^b^	1 (1.3)	1 (1.3)	1.00
LSIL ^c^	1 (1.3)	2 (2.6)	0.61
HSIL ^d^	1 (1.3)	0	1.00
Unsatisfactory	2 (2.6)	0	0.49
HPV PCR n (%) ^2^			0.13
Positive	15 (19)	8 (10.5)	
Negative	64 (81)	68 (89.5)	

^1^ Data presented as frequencies/proportions; Fisher’s exact test. ^2^ Data presented as frequencies/proportions; chi-square test. ^a^ Negative for intraepithelial lesion or malignancy. ^b^ Atypical squamous cells of undetermined significance. ^c^ Low-grade squamous intraepithelial lesion. ^d^ High-grade squamous intraepithelial lesion. HPV: human papillomavirus. PCR: Polymerase Chain Reaction.

**Table 3:** Cervicovaginal cytopathology and HPV types identified in 23 PCR-positive HTLV-1-infected and -uninfected women.

Sample ID	Cervicovaginal Cytopathology	HPV Sanger Types	HPV NGS Types
HTLV-1-infected (*n* = 15)			
3	NILM	53	Undetermined
12	NILM	58	Undetermined
13	NILM	58	Undetermined
15	NILM	Undetermined	42, 54, 70, 72
17	NILM	58	Undetermined
19	NILM	Undetermined	Undetermined
20	ASC-US	58	58
21	NILM	58	Undetermined
29	NILM	72	72, 74
37	NILM	58	58
41	NILM	Undetermined	26, 31, 68, 69
47	NILM	Undetermined	58
51	NILM	Undetermined	Undetermined
79	NILM	58	58
141	NILM	6	6, 16
HTLV-1-uninfected (*n* = 8)			
42	LSIL	61	Undetermined
83	LSIL	83	31, 35, 51, 59, 61, 83, 213
84	NILM	Undetermined	35, 44, 58, 59, 83, 84
92	NILM	71	Undetermined
102	NILM	Undetermined	Undetermined
120	NILM	33	33
126	NILM	Undetermined	54
144	NILM	61	Undetermined

NILM: negative for intraepithelial lesion or malignancy. ASC-US: atypical squamous cells of undetermined significance. LSIL: low-grade squamous intraepithelial lesion. HSIL: high-grade squamous intraepithelial lesion. HPV: human papillomavirus. PCR: Polymerase Chain Reaction. NGS: next-generation sequencing.

## Data Availability

The original contributions of this study are included in the article. Further inquiries can be directed to the corresponding authors.
